# An Energy Centric Cluster-Based Routing Protocol for Wireless Sensor Networks

**DOI:** 10.3390/s18051520

**Published:** 2018-05-11

**Authors:** A. S. M. Sanwar Hosen, Gi Hwan Cho

**Affiliations:** Division of Computer Science and Engineering, Chonbuk National University, Jeonju 54896, Jeonbuk, Korea; sanwar@jbnu.ac.kr

**Keywords:** wireless sensor network, clustering, routing, network lifetime

## Abstract

Clustering is an effective way to prolong the lifetime of a wireless sensor network (WSN). The common approach is to elect cluster heads to take routing and controlling duty, and to periodically rotate each cluster head’s role to distribute energy consumption among nodes. However, a significant amount of energy dissipates due to control messages overhead, which results in a shorter network lifetime. This paper proposes an energy-centric cluster-based routing mechanism in WSNs. To begin with, cluster heads are elected based on the higher ranks of the nodes. The rank is defined by residual energy and average distance from the member nodes. With the role of data aggregation and data forwarding, a cluster head acts as a caretaker for cluster-head election in the next round, where the ranks’ information are piggybacked along with the local data sending during intra-cluster communication. This reduces the number of control messages for the cluster-head election as well as the cluster formation in detail. Simulation results show that our proposed protocol saves the energy consumption among nodes and achieves a significant improvement in the network lifetime.

## 1. Introduction

Wireless sensor networks (WSNs) consist of sensor nodes with sensing and communication capabilities that have gained enormous attention for their usage in many applications, such as internet of things (IoT) [1,2]. Most wireless sensors are powered with batteries as limited energy sources. In addition, non-rechargeable energy sources strongly require minimizing energy consumption of the nodes and then maximizing network lifetime. Therefore, a lot of research has focused on energy conservation of sensor nodes in long-run operations of WSNs.

As a somewhat effective technique, clustering is an efficient way to save energy consumption of the sensor nodes. In the clustering process, sensor nodes are grouped into clusters. A node is selected as a cluster head, a leader in a cluster, and the remaining nodes within the cluster are considered cluster members. Sensor nodes sense the physical parameters related to their environment and send the information to their corresponding cluster heads. Cluster heads then aggregate the data and send it to a remote base station (BS) or sink using single-hop or multi-hop that depends on the distance of the BS.

Several cluster-based routing techniques can be found in [3,4] where periodic cluster head election and re-clustering is performed. In most of these techniques, cluster heads are elected either independently or based on competition of different weight functions. Similar cluster formations are followed by a joining message, either to a nearby cluster head or to a cluster head with a higher residual energy [5,6,7,8,9,10,11,12,13,14,15,16,17,18,19,20,21,22,23,24,25,26,27]. This can balance the energy consumption of the nodes but does not ensure the maximization of the steady state (the time the first node dies in the network) and the network lifetime as well. Because only the residual energy of a node is one of the key criteria to cluster head selection, not considering the average distance from member nodes does not guarantee that the energy consumption of the nodes is saved during intra-cluster communication. Moreover, periodic cluster-head election and re-clustering processes of the protocols require the broadcasting of control messages in each round, which is the energy waste of the power-limited sensor nodes. One solution is to piggyback the weight value of the nodes with the local data sent to a cluster head to hand over the role [26,27]. Thus, the number of control messages is minimized regarding the cluster-head elections throughout the network lifetime. But, consideration of energy saving in intra-cluster communication of the protocols does not exist yet.

In this paper, we propose an energy-centric cluster-based routing protocol called ECCR (energy-centric cluster-based routing) for WSNs that addresses the aforementioned issues. Firstly, we propose predefined static clusters that reduce the control message overhead of the cluster formation issue. Secondly, we introduce a caretaker for cluster-head election technique inspired by L. Malathi et al. [26], where the ranks’ information of the nodes are piggybacked along with the local data. A former cluster head from the previous round is responsible to hand over the role to a prospective cluster head in the current round. This reduces the control message overhead regarding the cluster head elections throughout the network lifetime. For data gathering and forwarding, the cluster heads are elected and selected based on the higher rank among the nodes. The rank of a node is defined by the factors which have a major influence on the energy consumption of the node. Experiments are performed on the proposed ECCR to compare with existing protocols such as EADUC, HUCL and IEADUC in [19,26,27], respectively. 

The rest of this paper is organized as follows. We review related works in Section 2. Section 3 presents the system model. The proposed protocol is described in Section 4. The results and comparison with existing protocols are given in Section 5, followed by the conclusion in Section 6.

## 2. Literature Review

Many routing protocols have been proposed for WSNs in the last decade [3]. It has been proven that distributed clustering with data-fusion technique is more energy-efficient when compared to other routing protocols [4]. Previous research works have been undertaken in the area of cluster-based routing algorithms. These clustering algorithms are commonly based on rotating the role of cluster heads and re-clustering in every round to prolong the network lifetime. The low energy adaptive clustering hierarchy (LEACH) protocol [5] is a pioneer work available in this category. The cluster head election process of LEACH is periodic and for every round, new cluster heads are elected. Although the LEACH protocol distributes the energy consumption among the nodes equally, it leads to additional routing overhead, resulting in excessive use of limited energy of a cluster head. Besides, the protocol assumes single-hop communication between the nodes and BS. It constructs the network by making it less scalable and is unsuitable for a large scale WSN.

LEACH gives birth to many protocols that have been developed in the last few years that improved over the network lifetime. The stable election protocol (SEP) has been proposed in reference [6] for heterogeneous WSN. It suggests that a percentage of nodes are equipped with more energy called advanced nodes compared to rest of the nodes. The advanced nodes create heterogeneity in terms of node’s initial energy. The cluster head election probabilities are weighted by the initial energy of a node relative to that of other nodes. This prolongs the steady state and the network lifetime as well. A new evolutionary-based routing protocol (ERP) has been proposed to extend the network lifetime [7]. It adopts a fitness function which incorporates two clustering aspects: intra-cluster distance in cluster formation and inter-cluster distance in clusters separation. ERP achieves better performance over SEP. S. Kumar et al. proposed an enhanced threshold-sensitive SEP (ETSSEP) [8]. It is based on dynamically changing the cluster-head election probability. ETSSEP selects cluster heads on the basis of residual energy level of nodes and minimum number of clusters per round. The election process also prolongs the network lifetime, which is longer than SEP. Besides, a multi-hop-based LEACH (M-LEACH) [9] and a distance-threshold-based cluster-head election on LEACH (LEACH-DT) [10] have been proposed. These two protocols achieved an enhanced network lifetime over LEACH. 

A hybrid energy-efficient distributed clustering (HEED) has been proposed by O. Younis and S. Fahmy [11]. It selects cluster heads according to the residual energy of nodes and intra-cluster distance as the primary and secondary criteria. Although it is successful in prolonging the network lifetime, it introduces an extra communication overhead to compute the communication cost with its neighbor nodes by exchanging a large number of control messages. Meanwhile, HEED may not be effective in load balancing as the nodes nearby to the BS die quickly. Many other clustering algorithms have also been proposed in the literature [12,13,14,15] which are similar to HEED and introduce high control messages overhead during cluster head selection and cluster formation. A distributed energy-efficient clustering (DEEC) protocol has been proposed by L. Qing et al. [16]. The authors proposed a cluster-head election method based on the ratio of residual energy of a node and the average energy of the network. Although, in all of these clustering and routing methods, a periodic rotation of cluster-head election based on different weight functions introduce the balanced energy consumption of nodes, none of these algorithms can take into account the “*energy hole*” problem in many-to-BS data-gathering.

Several methods have been proposed in literature to resolve this “*energy hole*” problem and thereby maximize the network lifetime. S. Soro and W. B. Heinzelman proposed an unequal clustering algorithm which was the first initiative regarding this issue [17]. It adopts unequal cirques called clusters where the clusters in the different cirques have different sizes. Some high-energy nodes can be deployed to take on and balance the energy consumption of these cluster heads. This can effectively prolong the network lifetime, but the position of cluster heads must be known previously. A further solution step has been taken through an energy-efficient unequal clustering (EEUC) mechanism [18]. It adopts a cluster-head election algorithm, where cluster heads are elected on the basis of residual energy of the nodes. A node is elected as a tentative cluster head with a probability *T* (a waiting time to broadcast an announcement message as a cluster head). Cluster heads use uneven competition ranges to form clusters of uneven sizes. The clusters nearby the BS have smaller sizes than the faraway clusters from the BS. The closer cluster heads consume less energy and preserve an amount of energy for the inter-cluster communication resulting in the balanced energy consumption of the cluster heads. Due to the quality of generated cluster heads being biased by the *T*, some nodes can be isolated in some cases of *T*. 

An energy-aware distributed unequal clustering (EADUC) algorithm has been proposed by J. Yu et al. that addresses the “*node isolation*” problem [19]. Here, a cluster head is elected based on the ratio of residual energy of a node and the average residual energy of its neighbor nodes. If a node does not receive any head message from the tentative and neighbor cluster head(s), the node elects itself as a cluster head and broadcasts the head message. The nodes will join with the nearby cluster head to form a cluster. A relay node is selected during inter-cluster communication on the basis of minimum distance towards the BS. This achieves an enhanced network lifetime by contrast with the previous protocols, but a relay node can be selected repeatedly due to the bias of the relay function. As a result, the node dies quickly. 

To resolve the issue of losing the nodes, an energy-aware routing algorithm (EADC) has been proposed in [20]. The proposed cluster-head election is similar to EADUC, but the relay function differs; instead, it uses a node’s residual energy and the number of member nodes as the key criteria to be a relay node. However, in the clusters’ formation of these algorithms [19,20], each node chooses the nearby cluster head in order to join without considering the residual energy of the cluster head. One of common problems in these techniques does not assure the relaying load of the cluster heads, which are balanced with respect to their residual energy. In other words, all the cluster heads are not participating in relaying the data for other cluster heads, so it results in an imbalanced energy consumption of the cluster heads, thus limiting the network lifetime. An energy-efficient multi-level and distance-aware clustering (EEMDC) has been proposed by A. Mehmood et al. [21] in the same regard. It divides the network into three logical levels that are based on the hop-count according to the BS. The levels are namely first-level clusters, second-level clusters, and third-level clusters that include nodes having a hop-count value of 1 to 2, 3 to 5, and 6 to more, respectively. Initially, the BS broadcasts a cluster-initiative packet of hop-count value over the network. A node receives the message and determines which cluster-level it belongs to. The nodes in different levels are determined by the distance of the BS. The number of nodes in a cluster and the number of clusters in different levels are varied due to the position of the nodes with respect to the BS. The proposed cluster head election is similar to LEACH’s. Unlike the first round as LEACH, the competition of a cluster head election is based on the residual energy and hop-count value of nodes from the next round. If there are multiple nodes with the same residual energy in a cluster, a node with the lower hope-count value is selected as a cluster head. The data-routing of EEMDC maintains a hierarchy from cluster heads to the BS according to the levels. This policy ensures a minimum distance route towards the BS. Meanwhile, the energy consumption of nodes is balanced and distributed over the network; thus, the network lifetime is enhanced over the protocols.

An energy and coverage-aware distributed clustering (ECDC) has been proposed in [22]. ECDC integrates the network coverage issue with the energy saving of nodes. The point and area coverages are studied along with the distributed routing. In both cases, the nodes are elected as cluster heads based on the residual energy and coverage importance metrics, respectively. The information of the metrics is shared among the neighbors within a range. The cluster head election process is similar to LEACH’s, where the minimum coverage importance and higher residual-energy-obtained nodes are elected as cluster heads. In ECDC, a data-routing algorithm is adopted where a relay node is selected based on the relay metric of the cluster heads towards the BS. The protocol achieves a lower energy consumption of nodes and better in-coverage performance compared to other protocols. 

An energy-aware routing algorithm (ERA) has been proposed by T. Amgoth and O. K. Jana [23] that addresses the problem of EADUC and EADC. In ERA, nodes consider a cluster head with higher residual energy to join in order to form a cluster. Unlike the relay functions of the route selection policies of the protocols, a directed virtual backbone (DVB) of the cluster heads has been adopted. The sink initiates broadcasting a route request message to the cluster heads. Cluster heads receive the message and increment its level to one higher than the sink (i.e., the level of the sink is assumed to be at zero, *L*(sink) = 0, so that *L*(*u*) = *L*(sink) + 1). Recursively, all the cluster heads broadcast the message to complete the process of forming a DVB. A cluster head selects the relay nodes based on the ratios of the average residual energy of the cluster heads in different levels and distributes all the aggregated data packets towards the sink sequentially. A decentralized energy-efficient hierarchical cluster-based routing algorithm (DHCRA) has been proposed by M. Sabet and H. R. Naji [24]. The key approach of the protocol scheme is that cluster heads are selected at the tree edges based on effective local information. The cluster heads selection is based on nodes’ residual energy and distance from the BS along with the constructed routing tree. This reduces a number of control messages and consequently saves energy consumption of the nodes. Hence, the network lifetime enhances, but the steady state of the network is not convincing. 

A distributed and adaptive routing protocol in cluster-based (DARC) routing has been proposed in [25]. The key approach of DARC is to adaptively adjust the routing mode of cluster heads to balance the energy consumption during inter-cluster communication. Even though the periodic clustering can distribute the energy consumption of cluster heads among all nodes, the imbalance in energy consumption exists due to the random location of nodes with respect to the distance between the BS. To address this problem, a relay cluster head is selected among the cluster heads on the basis of relay mode (i.e., CH-L and CH-H denote a cluster head with low and high energy) that is defined by the residual energy of the cluster heads. A CH-H selects the BS as its next hop and acts as a relay node for a CH-L. This process balances the energy consumption among the cluster heads by distributing the relay tasks to the CHs-H, which results in an improved network lifetime.

Unlike the typical cluster head election processes of the protocols, hybrid unequal clustering (HUCL) protocol suggests cluster head-hand-over and piggybacking techniques [26]. In the head-hand-over technique, a former cluster head delivers the role of cluster head to an elected node. In the piggybacking technique, the value of weight function is sent along with the local data to an associated cluster head. This reduces the control message overhead during cluster-setup phases. Hence, the steady state and the network lifetime are prolonged compared to other protocols. However, the number of neighbor nodes is not considered in defining competition radius along with the distance from the BS, although it uses the number of neighbor nodes while computing a waiting time to become a cluster head. To resolve this problem, an improvement of EADUC called IEADUC has been proposed in [27]. It combines the cluster head-hand-over technique of HUCL with the same cluster-head election technique of EADUC. Meanwhile, it modifies the relay function of a relay-node selection towards the BS. Unlike the EADUC, a relay node is selected on the basis of relay values defined by three factors; the residual energy of a cluster head, number of member nodes associated with the node, and the costs of data aggregation and transmission to a next hop. This achieves an enhanced network lifetime by contrast with the EADUC and HUCL. IEADUC can prolong the steady state and the network lifetime over the protocols. 

In all the algorithms, the cluster-head elections are based on different weight functions, where the factors are mostly residual energy of a node, number of neighbor nodes, and distance from the BS. But, in these weight functions, the intra-cluster communication distance has also not taken into consideration, whereas it has a significant impact on the overall energy consumption of the nodes. The cluster-heads election are biased by the time *T* and competition range of broadcasting a head message. Therefore, a cluster might have a less residual-energy-obtaining node as a cluster head compared to some of the member nodes, i.e., in [19,20,21,22,24,25,26,27]. On average, a high number of control messages is required to complete a single data transmission to the BS. Although some methods of the protocols are very effective to balance the energy consumption among the nodes, the control messages overhead use an excessive amount of energy of the nodes, which limits the network lifetime as a whole. Table 1 summarizes the comparison between the protocols on the basis of clustering, scalability, control messages overhead in clustering, and energy efficiency that results in steady state and network lifetime.

## 3. Preliminaries

### 3.1. Network Model and Assumptions

The network consists of *N* number of sensor nodes deployed randomly and uniformly in an *M* × *M* m^2^ sensor field. The nodes and BS are static after deployment. The nodes can be homogeneous or heterogeneous in terms of their initial energy. The nodes are location-aware, and each node has an identity (*id*) number. The nodes use power control to adjust the transmission power over a transmission distance.

### 3.2. Energy Consumption Model

The energy consumption is based on the first-order radio dissipation model [5]. The transmitter consumes energy *E*_TX_ in running the radio electronics and transmission amplifier circuitry. The receiver consumes energy *E*_RX_ in radio electronics. Depending on the transmission distance *d*, the free space *ϵ*_fs_ and multipath fading *ϵ*_mp_ channel models are used. The energy consumption of transmitting and receiving *l*-bit data over the distance *d* is according to Equations (1) and (2), respectively. A cluster head node consumes *E*_da_ (nJ/bit/signal) amount of energy in data aggregation and consumes *E*_com_ (nJ/bit/signal) in the processing of additional information of ranks and residual energy of associate nodes. We assume the sensed information by the nodes is highly correlated.

(1) $$E_{TX}(l,d)=\{\begin{array}{cc} l\times E_{elec}+l\times \varepsilon_{fs}\times d^{2} & if\;d\leq d_{0}\\ l\times E_{elec}+l\times \varepsilon_{mp}\times d^{4} & otherwise \end{array}$$

(2) $$E_{RX}(l,d)=l\times E_{elec}$$

where *E_elec_* is the power consumption for transmitting and receiving circuitry, a signal is amplified that depends on the distance *d*, and the reference distance *d*_0_ = √(*ε_fs_*/*ε_mp_*) = 87 m.

### 3.3. Data Aggregation Model

In this work, the infinite compressibility model [5] is used for data aggregation. It is assumed that a cluster head aggregates local data gathered from its members into a single packet of fixed length, irrespective of the number of received packets. The radio of each non-cluster head node is turned off until the allocated transmission time of node. The receiver of a cluster head node should be turned on to receive all the local data from the associate member nodes.

## 4. The Protocol Details

**Figure 1:**
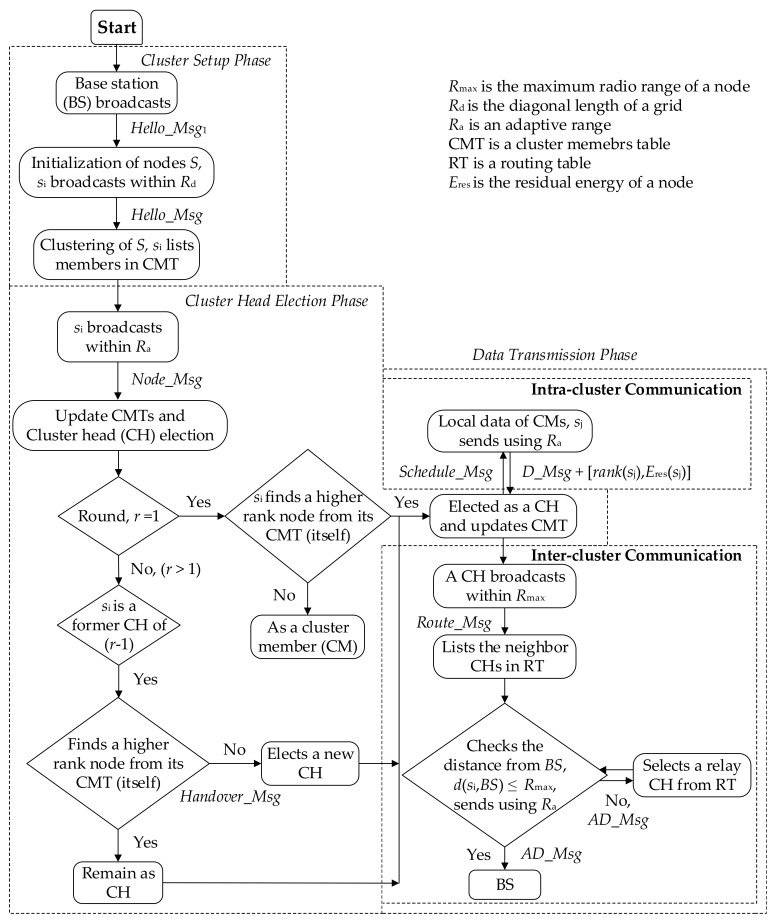
Flowchart of energy-centric cluster-based routing (ECCR) operation, including cluster setup, cluster-head election and data transmission.

The whole operation is divided into periodic rounds. Each round consists of the cluster head election and data transmission phases. Cluster members sense the information from the environment and send the collected data to their cluster head. A cluster head receives and aggregates the data from its member nodes and sends the data towards the BS based on the constructed routing path. Data transmission phase should be longer than cluster-head election phase to reduce the overhead of the protocol and to prolong the network lifetime. Several control and data messages are used. The description of the messages is given in Table 2. A flowchart of ECCR operation has been given in Figure 1.

### 4.1. Cluster Setup Phase

Generally, the number of clusters in a network may vary in several factors such as nodes’ energy, correlation of data, types of sensor nodes, size of the network, and the distance of the BS in different applications of WSNs [5,6,28,29,30]. Our proposed network is divided into a number of virtual grids. The nodes deployed in the grids are grouped into clusters that are static. Here, the number of clusters is defined as in Equation (3) according to [6]. The network deployment stage starts at time *T*_1_, where, the BS broadcasts a *Hello_Msg*_1_ to all the nodes _S_ = {*s*_1_, *s*_2_, *s*_3_, …, *s*_N_} at a certain power level. From the received signal of the message, each node *s_i_* can compute the approximate distance to the BS as *d*(*s_i_*,*BS*) based on the received signal strength [31]. Then each node broadcasts a *Hello_Msg* within the transmission range *R*_d_ (diagonal length of a grid) with the following two values: self *id* and cluster *id*. At the same time, it receives the *Hello_Msg*s from its neighbor nodes within the same cluster and updates its cluster members’ table CMT. The detail procedure of this phase is given by the pseudo-code in Algorithm 1.

(3) $$\#clusters=\sqrt{\frac{N}{2\pi }}\times \frac{M}{d_{avg}(CHs,BS)}$$

where *M* is the length of the sensor field, *d_avg_*(*CHs*,*BS*) is the approximate average distance from the prospective cluster heads to the *BS* and *CH* is denoted as cluster head.

**Algorithm 1.** Cluster Setup1: **start** (information collection)2:  **while** (*T*_1_ has not expired) **do**3:   *BS* broadcasts *Hello_Msg*_1_4:   Nodes receive the message5:   Measure distance from *BS*6:   *s_i_*.*state* ← *Normal*7:   Broadcast *Hello_Msg*8:   Receive and update CMT9:  **end**10: **end**

### 4.2. Cluster-Head Election Phase

During this phase, cluster heads are elected whose duration is *T*_2_. A cluster head is elected on the basis of rank of the member nodes in a cluster. Two different ways of cluster-head election technique are adopted. At the first round (*r* = 1), each node broadcasts a *Node_Msg* within its adjustable transmission range *R*_a_ (distance from a node to the farthest alive node in a cluster) with the following values: rank and residual energy. At the same time, every node in the same cluster receives the message and updates its CMT. A node calculates its *d_avg_* from other member nodes which are plugged-in during the node’s rank calculation as in Equation (4). We can see from the equation that a node with a higher residual energy and the minimum average distance from the member nodes has the higher rank. A node with the higher rank is elected as a cluster head. In a situation, if multiple nodes have the same rank, the node with the higher residual energy is selected as a cluster head. Once a node is elected as a cluster head, it sends the time-division multiple access (TDMA) schedule list to all its member nodes by broadcasting a *Schedule_Msg*.

$$d_{avg}=\frac{1}{m-1}\left(\sum_{j=1,i\neq j}^{m-1}d(s_{i},s_{j})\right)$$

where *m* is the number of member nodes in a cluster and *d*(*s_i_*,*s_j_*) is the distance between two member nodes *s_i_* to *s_j_*.

(4) $$rank(s_{i})=\frac{\alpha \times E_{res}}{E_{ini}}+\frac{\left(1-\alpha \right)}{d_{avg}}$$

where *α* is a weight factor interval of [0,1]. *E_ini_* and *E_res_* are the initial and residual energy of a node *s_i_*, respectively.

From the second round (*r* > 1), a cluster head is elected by the former cluster head of the previous round (*r*-1) in a cluster. Here, the role of a former cluster head is a caretaker for the current cluster-head election. It elects a node with a higher rank as a cluster head among the member nodes, where the rank value of the member nodes has been piggybacked along with the local data, which is sent by the member nodes during the intra-cluster communication. Then it sends a *Handover_Msg* to the elected node with the following information: cluster head *id* and missing node *id*. The elected node receives the message and updates its CMT and sends the TDMA schedule list to its member nodes by broadcasting a *Schedule_Msg*. Upon receiving the message, a member node updates its CMT. If a node has not sent its local data to its respective cluster head during the intra-cluster communication, it is considered a missing node or a dead node. A missing node will be excluded from the network and from the rest of the process. If no *Schedule_Msg* has been broadcasted from a cluster head within a predefined time, *Node_Msg*s are broadcasted among the member nodes followed by the competition of the cluster-head election that has been described as in the first round. Otherwise, a cluster head is elected by the former cluster head in a cluster recursively throughout the network lifetime. This phase is given by the pseudo-code in Algorithm 2.

**Algorithm 2.** Cluster Head Election1: **start** (cluster head election among nodes)2:  **while** (*T*_2_ has not expired) **do**3:   *state* ← *Candidate*4:   **if** (round, *r* == 1) **then**5:    Broadcast *Node_Msg*6:    Receive and update CMT[*s_i_*]7:    Find a higher rank node8:    *state* ← *Normal*9:    CMT[*s_i_*].*state* ← *Head*10:    Broadcast *Schedule_Msg*11:   **else if** (round, *r* > 1 && *s_i_* is a former cluster head of *r* − 1) **then**12:    Find a member node with a higher rank13:    Find missing node *id*14:    Send *Handover_Msg*15:    Find cluster head *id*16:    *state* ← *Normal*17:    CMT[*s_j_*].*state* ← *Head*18:    Broadcast *Schedule_Msg*19:    **if** (no *Schedule_Msg* has received) **then**20:     Repeat the cluster head election process as *r* ==121:    **end**22:   **end**23:  **end**24: **end**

### 4.3. Data Transmission Phase 

The process of data transmission to the BS is divided into two subphases: intra-cluster communication and inter-cluster communication, whose durations are *T*_3_ and *T*_4_, respectively. The subphases are subsequently described in the following subsections. The details of this phase are given by the pseudo-code in Algorithm 3.

**Algorithm 3.** Data transmission.1: **start** (cluster-based hierarchical routing)/* *intra-cluster communication*2:  **while** (*T*_3_ has not expired) **do**3:   Piggyback rank values along with local data to cluster heads4:   Cluster heads receive and aggregate the data5:  **end**/* *inter-cluster communication*6:  **while** (*T*_4_ has not expired) **do**7:   **if** (*s_i_*.*state* == ‘*Head*’) **then**8:    Compute the value of rank9:    Broadcast *Route_Msg*10:    Receive and update RT11:    **if** (*d*(*s_i_*,*BS*) ≤ *R*_max_) **then**12:     *nexthop* ← *BS*13:    **else**14:     Find a *CH* with a higher rank among less distant *CHs* from *BS*15:     *nexthop* ← *CH*16:     Send aggregated data17:    **end**18:   **end**19:  **end**20: **end**

#### 4.3.1. Intra-Cluster Communication

During intra-cluster communication, member nodes send the local data directly to their respective cluster head. A cluster head aggregates the received data from the associate member nodes. Each member node piggybacks the value of its rank and residual energy along with the local data.

#### 4.3.2. Inter-Cluster Communication

In inter-cluster communication, a cluster head forwards the aggregated data towards the BS. The route selection method to the BS is based on the rank of the cluster heads. For this, each cluster head calculates its rank by using Equation (5) and broadcasts its rank value and cluster head *id* along with a *Route_Msg* within *R*_max_, where *R*_max_ is the maximum transmission range of a node. On receiving the message, each cluster head updates its routing table RT. The ranking function guarantees that a cluster head with a higher residual energy, minimum number of member nodes, and minimum distance from the BS has the higher rank. If the BS is faraway from a cluster head (i.e., here, *d*(*s*_i_,*BS*) > *R*_max_), the cluster head selects one of the cluster heads with the higher rank and less distance from the BS from its RT. If multiple cluster heads have the same rank, the cluster head with the higher residual energy is selected as a forwarding node. A cluster head receives the data from other cluster heads, and it will forward the data directly to the next hop without aggregation.

(5) $$rank(CH)=\frac{E_{res}}{d(s_{i},BS)\times m\times E_{ini}}$$

where *s_i_* is an elected *CH*.

## 5. Performance Evaluation

In this section, the simulations are executed to evaluate the performance of ECCR. Firstly, we study how the parameters setting in ECCR impacts the network performance. Then, we examine the performance in terms of network lifetime. Besides, the steady state of the network has been taken into consideration. To validate the performance of ECCR, we compare the network lifetime with existing protocols EADUC, HUCL and IEADUC.

### 5.1. Simulation Setup

**Figure 2:**
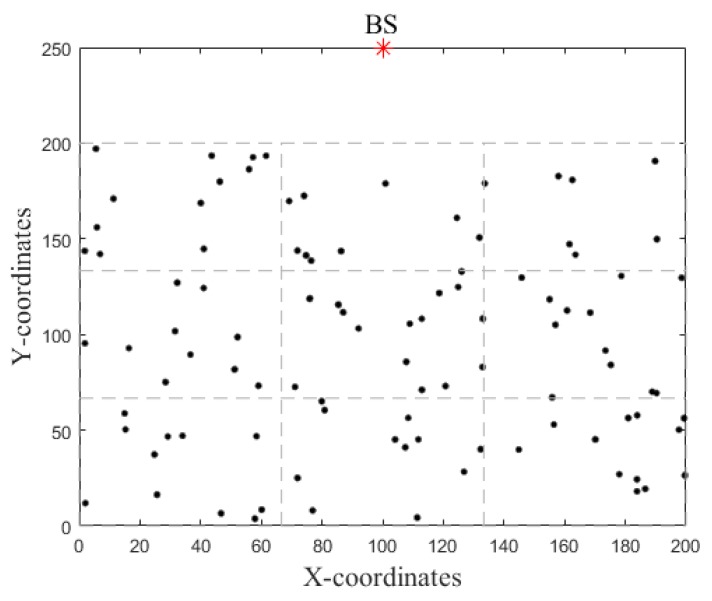
Example topology of the network.

The experiments were conducted in MATLAB R2017a. It is assumed that the location of the BS is set to (100,250) corresponding to the (*X*,*Y*)-coordinates of the sensor field. 100 nodes were randomly and uniformly deployed over a (200 × 200) m^2^ sensor field as shown in Figure 2. Nodes are heterogeneous and equipped with initial energy interval of 0.5–1.5 J. The rest parameters used in the simulations are given in Table 3. The other parameters used for the existing protocols are according to the protocols.

### 5.2. Network Lifetime

The network lifetime is defined as the number of rounds until a percentage of nodes die. The steady state is defined as the number of rounds until the first node dies. As the weight factor *α* has an important role in the network lifetime of ECCR, we first analyze the steady state and network lifetime of ECCR for various values of the weight factor.

**Figure 3:**
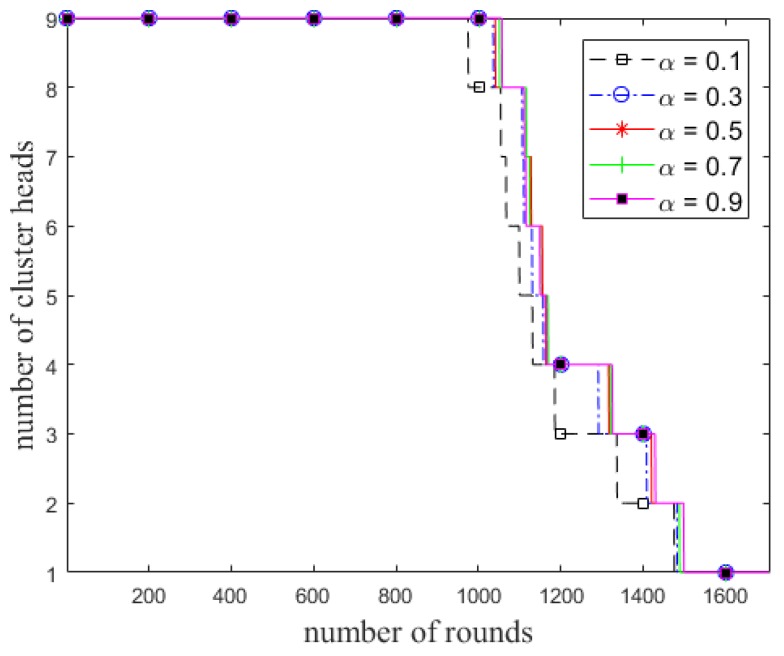
Number of elected cluster heads in each round in ECCR.

**Figure 4:**
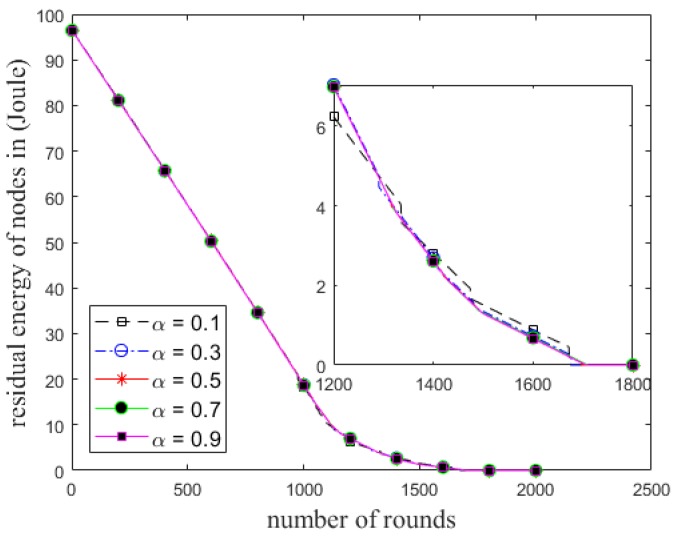
Total residual energy of the nodes in each round in ECCR.

**Figure 5:**
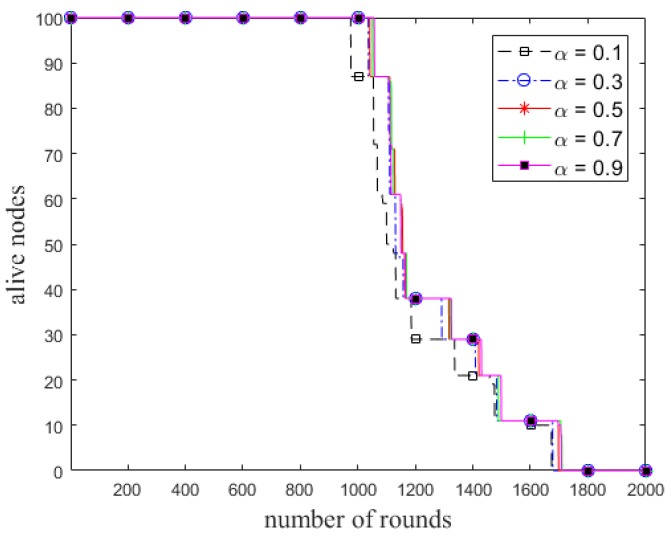
Number of alive nodes in each round in ECCR.

The number of cluster heads and total residual energy of the alive nodes in each round for different values of *α* in ECCR are shown in Figure 3 and Figure 4, respectively. It shows the number of alive clusters (all the nodes are being alive in the clusters) decreases due to increase in the number of rounds. The trend of the stability of a cluster being alive is proportional to *α*; this period of time is longer with a higher value of the weight factor. The total residual energy of the nodes is higher in each successive round with increases in the value of the factor. Figure 5 shows the network lifetime of ECCR varies depending on the different values of *α*. The results show that when the value of *α* is smaller, both the steady state and network lifetime are shorter. These metrics increase due to increasing the value of the factor in the same network circumstances.

**Figure 6:**
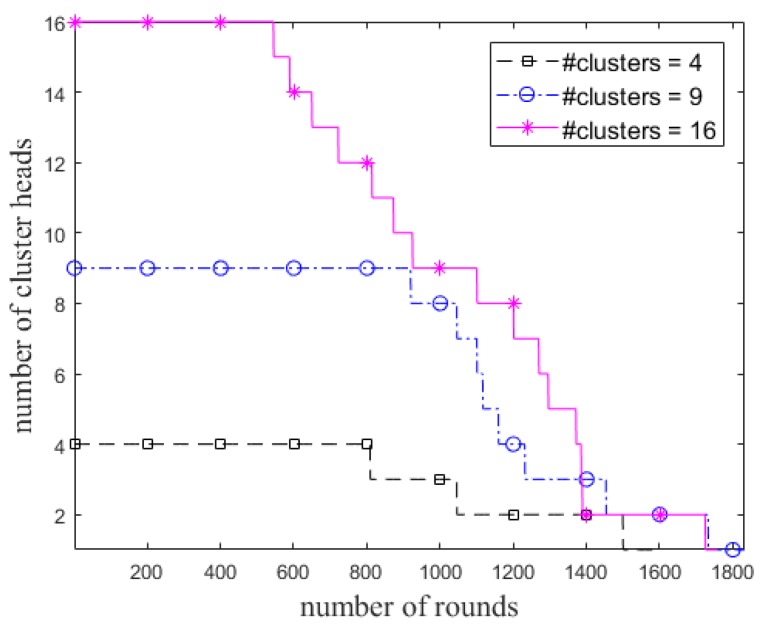
Number of elected cluster heads in each round in ECCR.

**Figure 7:**
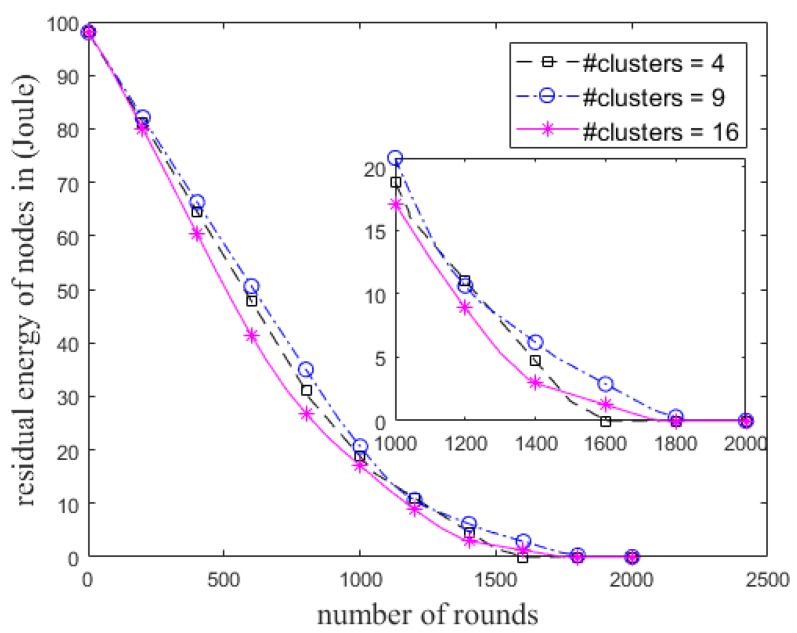
Total residual energy of the nodes in each round in ECCR.

**Figure 8:**
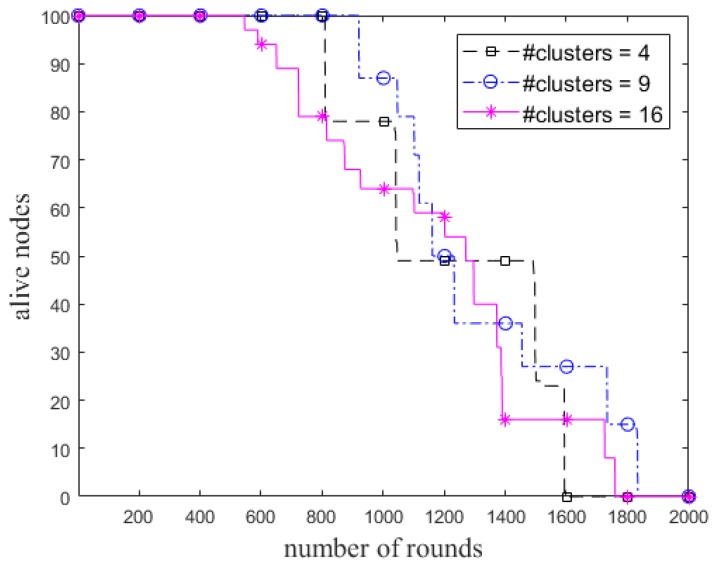
Number of alive nodes in each round in ECCR.

We also examine the ECCR for various lengths of the grid, so that the initial number of clusters is varied, as shown in Figure 6. Unlike the *R*_max_ of a node in the cases of 4 and 9 clusters, it was set to 2.5 × *R*_d_ for 16 clusters due to the position of the BS. The impact of total residual energy of the nodes in each round and the network lifetime are shown in Figure 7 and Figure 8, respectively. Figure 8 shows when *α* is constant (i.e., here *α* = 0.8), the steady state and network lifetime increase with increasing the number of clusters. These metrics increase until there are a certain number of clusters.

**Figure 9:**
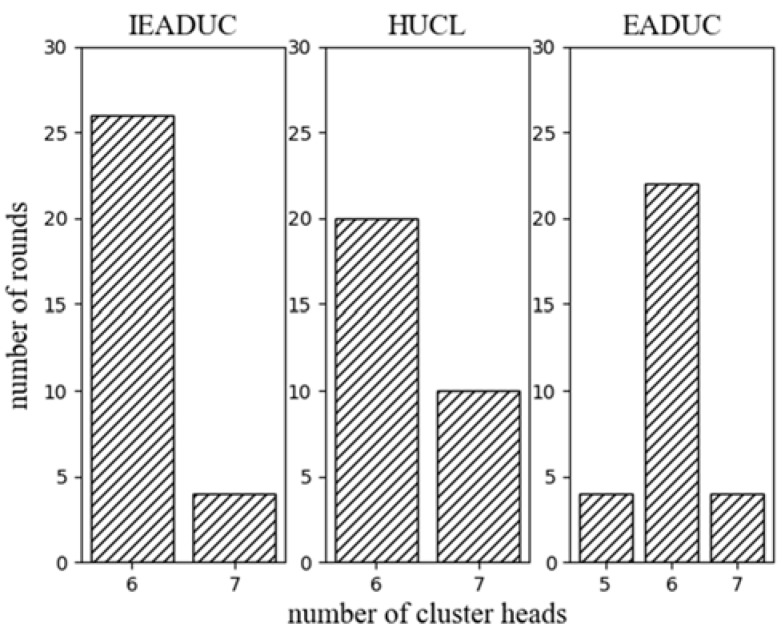
Distribution of the number of cluster heads.

Based on the earlier analysis of weight factor and number of clusters, we set the value of *α* = 0.8 and the initial number of clusters to 9 according to Equation (3). Then we evaluate the ECCR for the comparison of steady state and network lifetime with other protocols. The number of cluster heads in ECCR is predefined and static where it is varied in the protocols EADUC**,** HUCL and IEADUC. Figure 9 exhibits the distribution of the number of cluster heads in 30 randomly selected rounds of the protocols. The protocols control the competition radius and ensure that in each competition range, there is a cluster head. Therefore, the number of the cluster heads is well distributed across the network throughout their network lifetime. The trends of the energy consumption of the nodes in the protocols are distinctive. Trends vary according to their cluster head elections and data transmission to the BS.

**Figure 10:**
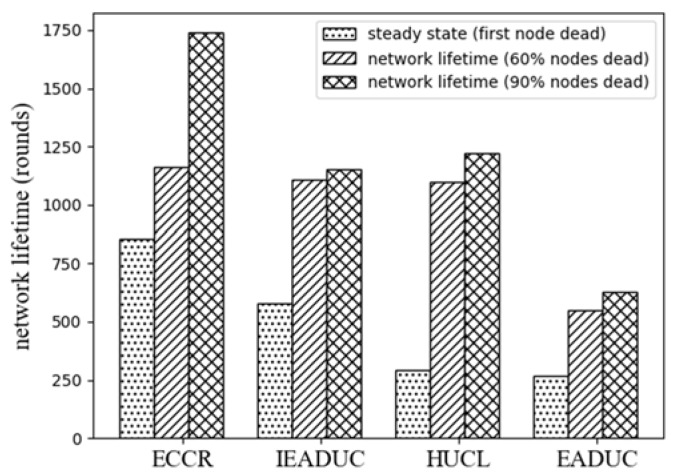
Network lifetime.

Figure 10 shows the ECCR outperforms the protocols in terms of steady state and network lifetime. The ECCR achieves on average of 3.16 times, 2.94 times and 1.47 times of steady state of the protocols EADUC, HUCL and IEADUC, respectively. The network lifetime considering 60% of the nodes are dead, ECCR enhances the network lifetime on average of 2.10, 1.05 and 1.04 times of the protocols, accordingly. The network lifetime of ECCR enhances significantly with increases of the percentage of dead nodes. ECCR achieves on average of 2.76, 1.42 and 1.51 times of the protocols considering 90% of the nodes are dead in the network.

### 5.3. Discussion

The reasons for the superior performance in network lifetime of ECCR are justified and summarized as follows.

- Periodic clustering approach of the existing protocols requires an additional energy consumption of the nodes for broadcasting and receiving a number of control messages in every round. Unlike re-clustering of the protocols, the static clusters of ECCR do not require any control message after the initial clusters formation. Hence, this saves the energy consumption of the nodes in this regard for rest of network operation.
- The cluster head elections of EADUC, HUCL and IEADUC are based on the residual energy of nodes where the average distance among the neighbor nodes has not taken into consideration. Only the residual energy of a node might not be an effective factor to select a cluster head, even though a node with a higher residual energy can sustain as a cluster head for a longer period of time. In these protocols, a node with a higher residual energy as a cluster head does not guarantee the minimum amount of energy consumption during the intra-cluster communication. It may happen due to the higher communication distance between the cluster head and member nodes. Meanwhile, the common cluster head election process of the protocols does not guarantee that only the higher residual-energy-obtained nodes are elected as the cluster heads across the network. Some of the cluster heads might have less residual energy compared to their member nodes due to the biased time *T* and cluster formation policies. Thus, the energy consumption among the nodes is not properly balanced, and some nodes may die quickly as a result. In contrast, our proposed cluster-head election technique is based on the ranks of the nodes and considers the residual energy and average distance among member nodes as well. Along with the balancing energy consumption of the nodes, the proposed method saves the energy of the nodes during intra-cluster communication over the network. Moreover, the piggyback and caretaker techniques in cluster-head election reduce a significant number of control messages throughout the network lifetime as in HUCL and IEADUC.
- A shortest route considering only the distance from a cluster head to the BS for data forwarding might cause the packets dropped of aggregated data packets at a certain relay node due to the lack of residual energy of the node to process the data and transmits to the next hop towards the BS, such as in EADUC. Distributing a load of aggregated packets across the network might not only increase the number of hops but also might consume an additional amount of energy of the associated cluster heads in the routes. It impacts on the overall network lifetime, such as in EADC. Instead, our adopted route selection towards the BS constructs a preferable energy-efficient route. It associates the cluster heads with higher ranks. The selection process considers the residual energy, number of member nodes, and distance from the BS. The method balances the energy consumption of the cluster heads as well as minimizes the number of average hops, which is more energy efficient in this regard.

## 6. Conclusions

In this paper, an energy-centric cluster-based routing protocol for wireless sensor networks is proposed. A static clustering is adopted, so that the deployed nodes can save energy, unlike the periodic clustering throughout the network lifetime. The cluster-head election is weighted on the basis of ranks that consider the residual energy and average distance of nodes. This balances the energy consumption of nodes along with saving the energy in intra-cluster communication. Moreover, the adopted piggybacking and hand-over techniques reduce a significant number of control messages regarding the cluster-head competition in each round. Besides in routing, the ECCR consider the major factors of energy consumption, which balances the energy among the cluster heads. It also ensures an energy-efficient route towards the BS. Overall, the proposed algorithm takes into consideration that saving the energy of nodes can achieve and sustain a prolonged network lifetime.

An extensive simulation is performed to evaluate the performance of ECCR. The selected performance metrics for this analysis is mainly the steady state and network lifetime. The simulation results validate ECCR protocol performed better for the selected metrics as compared to the existing protocols. 

## Figures and Tables

**Table 1:** The metrics and performance of the protocols.

Protocol	Clustering	Scalability (Single-Hop or Multi-Hop)	Control Message Overhead	Steady State of the Network	Network Lifetime
LEACH	dynamic	single-hop	medium	very low	very low
SEP	√	√	√	low	medium
ERP	√	√	√	√	√
ETSSEP	√	√	√	medium	√
M-LEACH	√	multi-hop	√	low	low
LEACH-DT	√	√	√	√	medium
HEED	√	√	high	√	low
DCAAB [12]	√	single-hop	√	√	√
GESC [13]	√	multi-hop	√	√	medium
ELBC [14]	static	single-hop	√	medium	high
ALBC [15]	√	√	√	√	√
DEEC	dynamic	√	medium	low	low
PLUC [17]	√	multi-hop	√	√	√
EEUC	√	√	√	medium	√
EADUC	√	√	high	√	medium
EADC	√	√	√	√	√
EEMDC	hybrid	√	√	√	√
ECDC	dynamic	√	√	√	√
ERA	√	√	medium	√	high
DHCRA	√	√	√	√	√
DARC	√	√	√	√	medium
HUCL	hybrid	√	low	high	high
IEADUC	√	√	√	√	√

**Table 2:** Description of the control and data messages.

Message	Description
*Hello_Msg*	Tuple (selfid, clusterid), a control message of nodes’ initial information.
*Node_Msg*	Tuple (selfid, selfrank, selfenergy), a control message of members’ information.
*Handover_Msg*	Tuple (selfid, clusterid, headid), a control message of hand over the role of cluster head to a prospective cluster head in a cluster.
*Schedule_Msg*	Tuple (schedule, order), a control message for assign the time slot for a member node to send local data to an associate cluster head.
*Route_Msg*	Tuple (selfid, selfrank, selfenergy, disttoBS), a control message to collect neighbor cluster heads’ information.
*D_Msg*	Tuple (selfid, clusterid, selfrank, selfenergy, ‘*local data*’), a local data message from a member node to associate cluster head.
*AD_Msg*	Tuple (selfid, clusterid, nexthopid, ‘*fused data*’), an aggregated data message from a cluster head to a next hop or BS.

**Table 3:** Simulation parameters setting.

Parameter	Value
Location of the BS	(100,250) m
Number of nodes, *N*	100
Initial energy, *E_ini_*	0.5–1.5 J
Control packet size, *l*	25 bytes
Data packet size, *l*	500 bytes
Transmitter or receiver circuitry, *E_elec_*	50 nJ/bit
Data aggregation cost, *E_da_*	5 nJ/bit/signal
Computation cost of rank and energy, *E_com_*	5 nJ/bit/signal
Transmit amplifier cost, *ε_mp_*, if (*d* > *d*_0_)	0.0013 pJ/bit/m^4^
Transmit amplifier cost, *ε_fs_*, if (*d* ≤ *d*_0_)	10 pJ/bit/m^2^
Diagonal length of a grid, *R*_d_	√2 × (*M*/3) = 94.28 m
Maximum transmission range, *R*_max_	2 × *R*_d_ = 188.56 m
Weight factor, *α*	0.8
